# Acute care utilization among individuals with sickle cell disease and related cardiopulmonary and renal complications

**DOI:** 10.1371/journal.pone.0297469

**Published:** 2024-04-16

**Authors:** Ashima Singh, David C. Brousseau, Mahua Dasgupta, Arun S. Shet, Joshua J. Field, Amanda M. Brandow

**Affiliations:** 1 Department of Pediatrics, Medical College of Wisconsin, Milwaukee, Wisconsin, United States of America; 2 Department of Pediatrics, Nemours Children’s Health System, Wilmington, Delaware, United States of America; 3 Laboratory of Sickle Thrombosis and Vascular Biology, Sickle Cell Branch, National Heart, Lung, and Blood Institute, National Institutes of Health, Bethesda, Maryland, United States of America; 4 Department of Medicine, Medical College of Wisconsin, Milwaukee, Wisconsin, United States of America; 5 Versiti Blood Center of Wisconsin, Milwaukee, Wisconsin, United States of America; University of Illinois at Chicago, UNITED STATES

## Abstract

Cardiopulmonary and renal end organ (CPR) complications are associated with early mortality among individuals with sickle cell disease (SCD). However, there is limited knowledge regarding acute care utilization for individuals with SCD and CPR complications. Our objective was to determine the prevalence of CPR complications in a state specific SCD population and compare acute care utilization among individuals with and without CPR complications. We leveraged 2017–2020 data for individuals with SCD identified by the Sickle Cell Data Collection program in Wisconsin. The prevalence of CPR complications is determined for distinct age groups. Generalized linear models adjusted for age compared the rate of acute care visits/person/year among individuals who had cardiopulmonary only, renal only, both cardiopulmonary and renal, or no CPR complications. There were 1378 individuals with SCD, 52% females, mean (SD) age 28.3 (18.5) years; 48% had at least one CPR complication during the study period. The prevalence of CPR complications was higher in adults (69%) compared to pediatric (15%) and transition (51%) groups. Individuals with SCD and cardiopulmonary complications had higher acute visit rates than those without CPR complications (5.4 (IQR 5.0–5.8) vs 2.4 (IQR 2.1–2.5), p <0.001)). Acute care visit rates were similar between individuals with SCD who had renal only complications and no CPR complications (2.7 (IQR 2.5–3.0) vs 2.4 (2.1–2.5), p = 0.24). The high acute care visit rates, especially for those with cardiopulmonary complications, warrant further investigation to understand risk factors for CPR complications, the underlying reasons and identify effective disease management strategies.

## Background

Sickle cell disease (SCD) directly affects the hemoglobin in red blood cells, but the cascade from those effects makes individuals with SCD at increased risk for acute and chronic end organ damage impacting the heart, lung and kidneys [1, 2]. The cardiopulmonary and renal (CPR) end organ complications among individuals with SCD are associated with early mortality [3–5], however data evaluating acute care utilization for individuals with SCD and CPR complications are lacking. Cardiopulmonary and renal damage can occur due to common mechanisms of chronic hemolysis, and repeated sickling episodes that promote microvascular alteration among individuals with SCD. Further, bidirectional interactions between renal and cardiopulmonary disease can further exacerbate CPR end organ complications. For example, chronic kidney failure, in a vicious cycle, can lead to progressive myocardial and vascular damages. Further, cardiopulmonary complications in individuals with SCD may be related to the presence of nephropathies [6]. The understanding of the proportion of the SCD population that has both of these complications is limited. Also, individuals with SCD have high rates of acute care utilization; with acute painful events being the leading cause of these visits. However significant variation exists in acute care use between individuals [7, 8]. It is not known whether this variation in the rate of SCD acute care visits could be driven by those who have CPR end organ complications. Due to the association of CPR complications with mortality, it is conceivable that acute care utilization could be higher in those with CPR complications. Knowledge of the acute care utilization for people with SCD and CPR complications is needed to better understand the heterogeneity of the impact of SCD on patients’ lives and the health care system. Further, this knowledge could help clinicians determine if alternate SCD care models are needed for those with CPR end organ complications.

The objective of our study was to compare the rate of acute care utilization (all cause and that for acute pain crises) between individuals with SCD with and without CPR end organ complications using data from a Centers for Disease Control and Prevention (CDC) funded surveillance program for SCD in Wisconsin [9]. We hypothesized that individuals with SCD who have CPR complications will have significantly higher rates of acute care utilization compared to those who do not have CPR complications.

## Methods

### Data source

#### Data from statewide surveillance program in Wisconsin

We leveraged the Sickle Cell Data Collection program in Wisconsin (SCDC-WI). The goal of this CDC funded program is to study long-term trends in diagnosis, treatment, and healthcare access for people with SCD in Wisconsin. This program aims to include all individuals living with SCD in Wisconsin. The SCDC-WI, as of December 2022, includes data from newborn screening (2013–2020), Medicaid (2018–2020), other health insurance data available via the Wisconsin Health Information Organization (2017–2020), electronic health records (EHR) data from two large tertiary care facilities (2013–2020), and hospital and emergency department discharge data from the Wisconsin Hospital Association (2013–2020).

Individuals were considered to have SCD if they had a confirmed newborn screening SCD result; or three or more visits or claims, regardless of location or type of service, with an associated diagnosis code of SCD during a 5-year time period or the duration of available data if less than 5 years. This definition has demonstrated high performance with ~90% positive predictive value for pediatric and adult individuals with SCD [10, 11]. Further, to increase the specificity of the SCD cohort, and consistent with previous publications, we excluded individuals who had a greater number of visits or claims with a sickle cell trait diagnosis than those with a SCD diagnosis [12, 13]. This would minimize the misclassification of those with sickle cell trait as having sickle cell disease. This study included individuals with SCD who met the above definition in the SCDC-WI program and had at least one claim or visit during the years 2017–2020.

The identification of SCD-related CPR end organ complications for inclusion were identified through consultation with pediatric and adult hematologists with expertise in SCD-related cardiopulmonary and renal complications and by literature review [4, 14–17]. We shared a list of complications with these experts and sought active feedback to revise the list and identify the ones that can be a result of the underlying condition of SCD. Individuals were considered to have cardiopulmonary complications if they had an ICD diagnosis code for cardiomyopathy or heart failure, dysrhythmia, pulmonary hypertension, long QT syndrome or pulmonary fibrosis. Renal complications included an ICD diagnosis code for chronic kidney disease (including end stage renal), proteinuria, or hematuria during the study period. The ICD codes are provided in the S1 Table.

### Outcome(s) of interest

Our primary outcome was the rate of acute care visits (including emergency department (ED) treat-and-release) and hospitalizations for any cause. The secondary outcome was acute care visits for pain crises.

The acute care visits were determined for the cohort of individuals who had at least one year of data during the observation period. The duration of available data was determined as the time difference between the first and the last encounter during the observation period. The type of acute care visit was based on the place of service assigned for the encounter. Specifically, Centers for Medicaid and Medicare Services (CMS) place of service codes of 21 and 23 were used to identify hospitalizations and ED visits respectively within the claims data. The CMS maintain the place of service code to be used throughout the healthcare industry in the nation and is often needed to determine the acceptability of billing services. The encounter type in the EHR data from are in the i2b2 common data model format, and the type of encounter was identified based on the values for encounter type. Any identified ED visit that resulted in hospitalization on the same day or within one day was considered as a hospitalization instead of a treat-and-release visit. An acute care visit was considered to be for a pain crisis if it had an associated diagnosis code for SCD crisis, unspecified. The acute care visits that only included codes for crises with acute chest syndrome, splenic sequestration or cerebral vascular involvement were not included as pain crises visits.

### Analysis

We determined the prevalence of the CPR end organ complication within the identified cohort by calculating the proportion of people with a diagnosis code of one or more of the specified CPR end organ complications at any time during the study period. The prevalence between distinct age groups and sex was compared using chi square tests. Individuals less than 18 years of age at the end of the study period were classified as pediatric group, 18–25 years were transition group, and those greater than 25 years of age were included in the adult group.

The rate of acute care visits was determined as the number of acute care visit per year per person. To calculate rates, the observation time for each individual was calculated as the difference in dates between the first and last visit or claim (any type) during the study period. The rate of both all cause and pain crises acute care visits (including ED and hospitalizations) were compared between individuals with SCD with and without CPR end organ complications using generalized linear models assuming negative binomial distribution adjusted for age. False discovery rates were used to adjust for multiple comparisons between groups. P-value of <0.05 was considered significant. This study used existing surveillance data, was deemed not research by the IRB, and therefore was exempt from IRB review at the Medical College of Wisconsin. The IRB waived the requirement for informed consent for the surveillance program and subsequently these analyses and publication.

## Results

There were 1378 individuals identified as having SCD in Wisconsin who had an encounter or claim during the years 2017–2020 (Table 1). The mean age of the identified cohort was 28.3 (standard deviation, sd = 18.5) years and 19.5% were older than 45 years. Those with CPR complications were significantly older than those without CPR complications (mean age 37.6 (sd = 16.1) years versus 19.7 (sd = 16.2) years, p<0.001). There were no significant differences in sex within the state between those who had CPR complications and those who did not.

**Table 1:** Demographic and clinical characteristics of the individuals with SCD with and without cardiopulmonary complications.

	N (%)	SCD with CPR	SCD with no CPR	p
	(N = 1378)	(N = 663)	(N = 715)	
Age based groups				
Years ≤18	485 (35.2)	73 (11.0)	412 (57.6)	<0.0001
Years >18 and < 25	152 (11.0)	78 (11.8)	74 (10.3)	
Years ≥25	741 (53.8)	512 (77.2)	229 (32.0)	
Mean (SD) age	28.3 (18.5)	37.6 (16.1)	19.7 (16.2)	<0.0001
Sex, Female	723 (52.5)	362 (54.6)	361 (50.5)	0.13
Genotype				
Region of WI				
Southeastern	1099 (79.8%)	547 (82.5%)	552 (77.2%)	0.13
Southern	140 (10.2%)	64 (9.7%)	76 (10.2%)	
Northeastern	69 (5.0%)	25 (3.8%)	44 (6.2%)	
Western	18 (1.3%)	<10	12 (1.7%)	
Northern	<10	<10	<10	
Missing/Out of state zip	46 (3.3%)	18 (2.7%)	28 (3.9%)	
USDA 2013 Rural Urban Continuum Codes**				
Metro	1301 (94.4%)	630 (95.0%)	671 (93.8%)	>0.9
Non metro	31 (2.2%)	15 (2.3%)	16 (2.2%)	
Missing/Out of state zip	46 (3.3%)	18 (2.7%)	28 (3.9%)	

### Prevalence of diagnosed CPR complications

**Fig 1:**
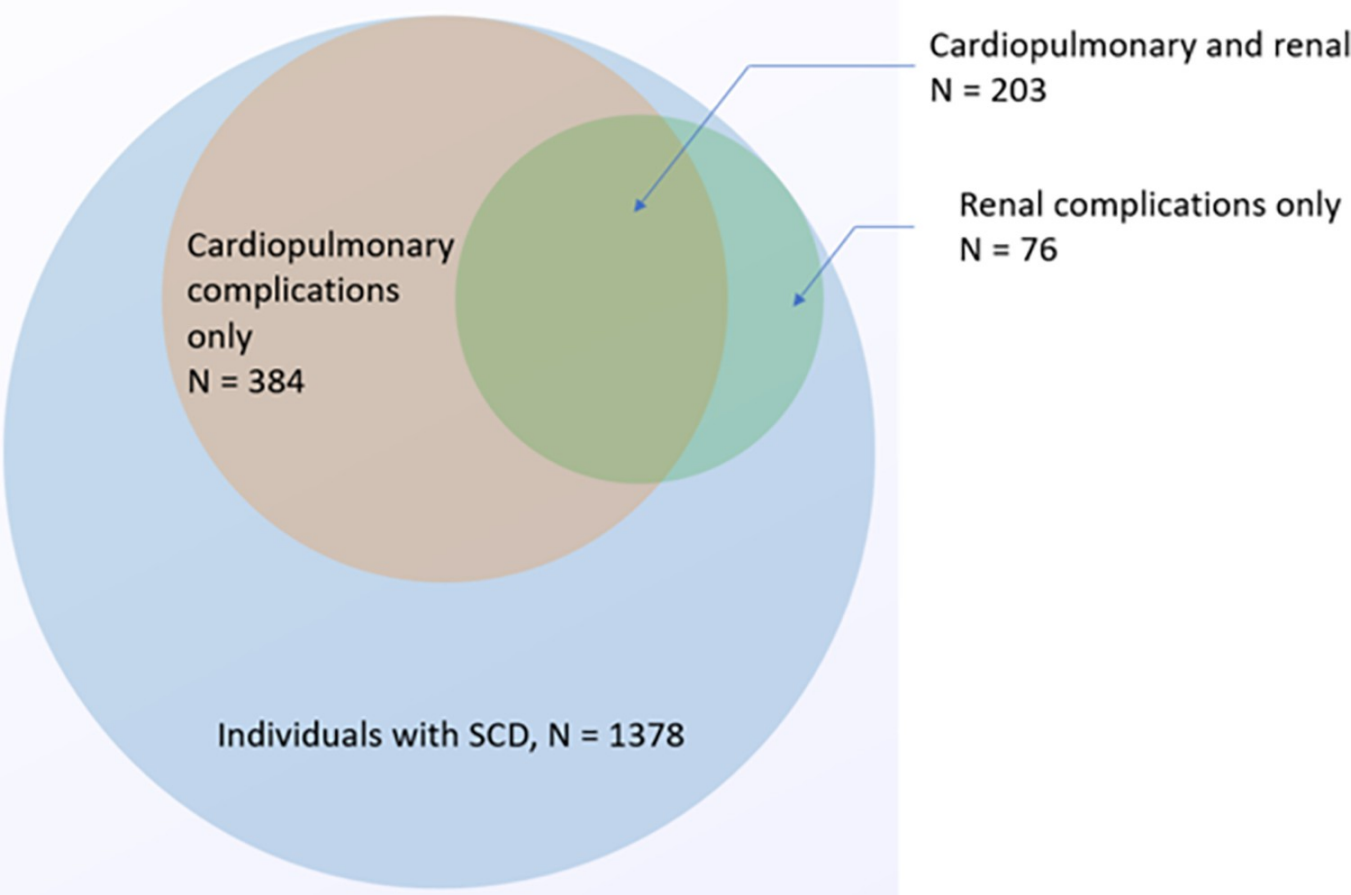
Venn diagram showing the prevalence and overlap of cardiopulmonary and renal complications among individuals with SCD living in Wisconsin. Each circle depicts the number of individuals with the respective cardiopulmonary or renal complications among the SCD population. The overlap between circles indicates the presence of both conditions.

Overall, 48% of the cohort had at least one diagnosis of a CPR complication. Fig 1 shows the prevalence and overlap of the cardiopulmonary and renal complications among the SCD population. There were 15% (n = 203) who had both cardiopulmonary and renal complications, 28% (n = 384) who had only cardiopulmonary complications, and 6% (n = 76) who had only renal complications. Of all the complications considered, dysrhythmia was most observed. Adults had a higher prevalence of CPR complications compared to those in the pediatric and transition age groups (69% of adults vs 51% of transition; 69% of adults vs 15% of children). Specifically, adults (63%) had a significantly higher prevalence of cardiopulmonary complications compared to those in the transition (45%) and the pediatric (10%) age groups. Individuals with SCD in the transition group (20%) and adults (29%) also had a higher prevalence of renal complications as compared to the pediatric (6%) age groups.

The number of individuals with each condition included in the cardiopulmonary and renal complication categories are shown in Table 2.

**Table 2:** Number of individuals with SCD in the state of Wisconsin having cardiopulmonary and renal complications.

	All		Pediatric		Transition		Adults	
			(0 –<18 years)		(18–25 years)		(>25 years)	
	(N = 1378)		(N = 485)		N = 152)		(N = 741)	
	N	%	N	%	N	%	N	%
Cardiopulmonary complications	587	42.6	50	10.3	68	44.7	469	63.3
Heart failure	151	11.0	<15	-	<15	-	146	19.7
Cardiomyopathy	49	3.6	<15	-	<15	-	44	5.9
Dysrhythmia	480	34.8	42	8.7	62	40.8	376	50.7
Pulmonary hypertension	190	13.8	<15	-	<15	-	170	22.9
Long QT	80	5.8	<15		<15	-	70	9.4
Pulmonary fibrosis	42	3.0	<15		<15	-	40	5.4
Renal complications	279	20.2	28	5.8	31	20.4	220	29.7
Chronic kidney disease	139	10.1	<15	-	<15	-	134	18.1
Proteinuria	76	5.5	15	3.1	<15	-	51	6.9
Hematuria	123	8.9	<15	-	22	14.5	90	12.1

### Acute care utilization: All cause

Eighty-nine percent (n = 1226) of the identified SCD cohort during 2017–2020 had at least one year of data during the observation period. This subset had 18,412 acute care visits (5,632 hospitalizations and 12,780 ED treat and release visits) over the four years. Overall, an individual with SCD in Wisconsin had a median of 2 acute care visits (IQR = 1–4) per year.

**Fig 2:**
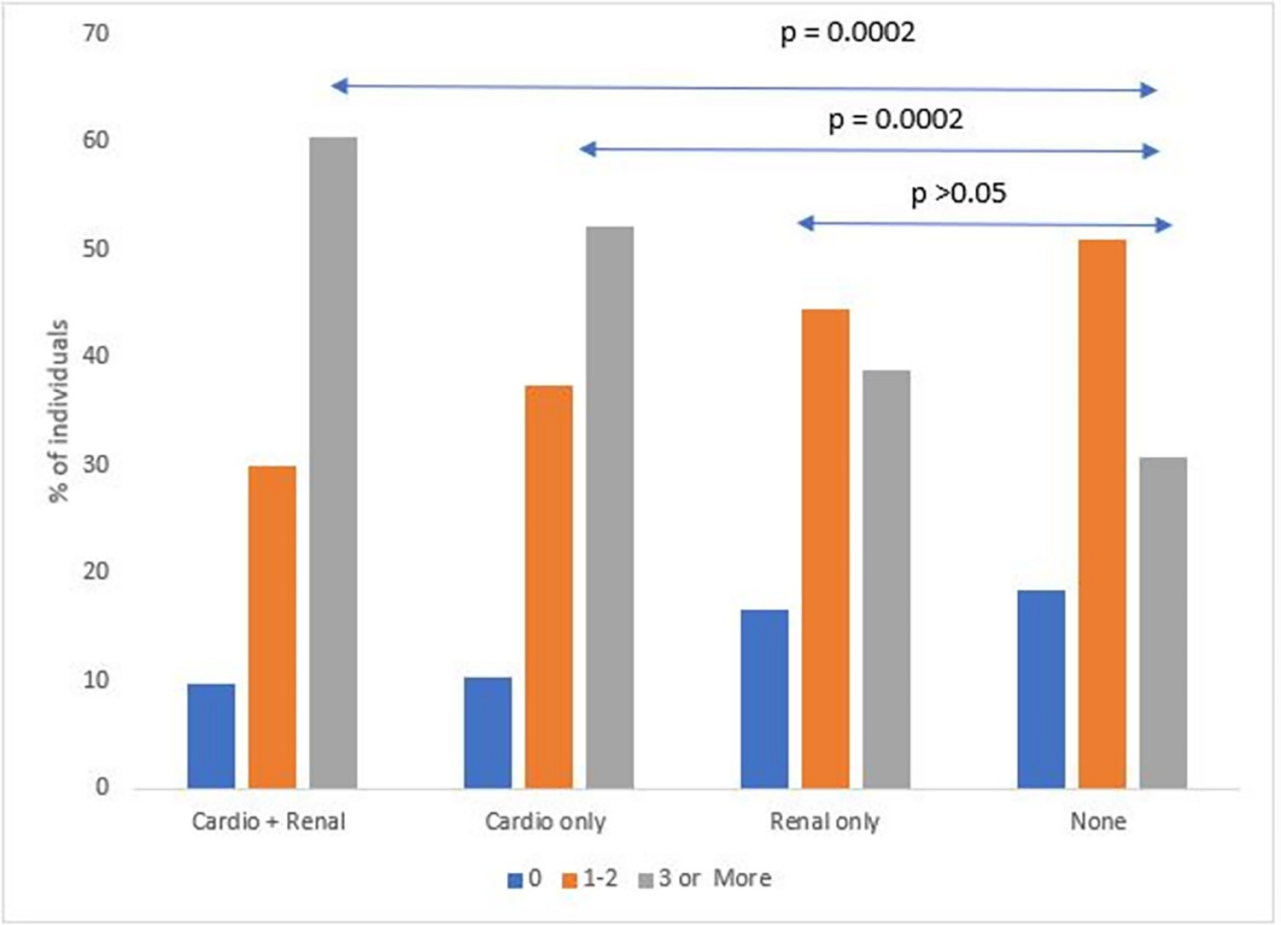
All-cause acute care visits by CPR groups. The box plots illustrate distribution of acute care visits per person per year for the four mutually exclusive groups of individuals with SCD with outliers depicted as circles. The label “Cardio + Renal” indicates the group with individuals who have both cardiopulmonary and renal complications; “Cardio only” indicates individuals with cardiopulmonary but no renal complications, “Renal only” indicates individuals with renal but no cardiopulmonary complications and “None” indicates individuals with neither cardiopulmonary nor renal complications.

There were 14.5% who had 0 acute care visits, 43.1% had 1–2 and 42.3% had 3 or more acute care visits per year. The distribution of acute care visits per person per year for those with cardiopulmonary, renal, both cardiopulmonary and renal, and neither is shown in Fig 2. A significantly higher proportion of individuals with only cardiopulmonary complications had 3 or more acute care visits in a year (52%) as compared to those who did not have any CPR complications (30.6%), p = 0.0002. However, this distribution was not significantly different between those with only renal complications (38.9%) and those with no CPR end organ complications (30.6%), p = 0.51.

After adjusting for age and multiple comparisons, those with CPR complications had a significantly higher rate of all cause acute care visits as compared to those without a diagnosis of CPR complications (median acute care visits per person per year 5.2 (IQR = 4.7–5.5 vs 2.4 (IQR 2.1–2.5), p <0.001).

Specifically, those with cardiopulmonary complications (including only cardiopulmonary complications, and those with both cardiopulmonary and renal complications) had a significantly higher rate of acute care use compared to those who did not have CPR complications (5.4 (IQR = 5.0–5.8) vs 2.4 (IQR = 2.1–2.5), p <0.001)). However, there were no differences in the rates of acute care use between individuals with renal complications only and those with no CPR complications after adjusting for age (2.7 (IQR = 2.5–3.0) vs 2.4 (2.1–2.5), p = 0.24).

### Acute care utilization for pain crisis

**Fig 3:**
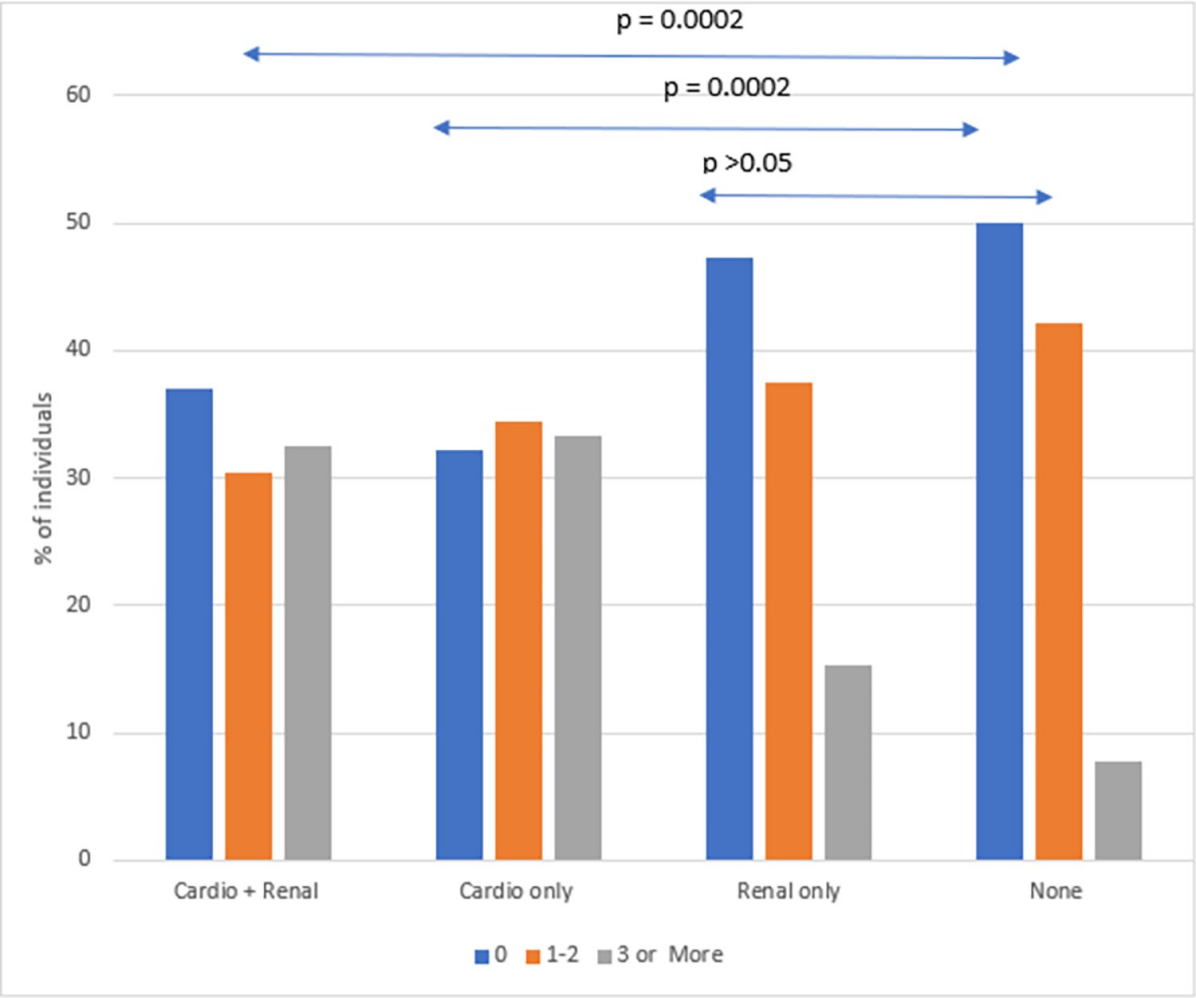
Pain crises acute care visits by CPR groups. The box plots illustrate distribution of acute care visits per person per year for the four mutually exclusive groups of individuals with SCD with outliers depicted as circles. The label “Cardio + Renal” indicates the group of individuals who have both cardiopulmonary and renal complications; “Cardio only” indicates individuals with cardiopulmonary but no renal complications, “Renal only” indicates individuals with renal but no cardiopulmonary complications and “None” indicates individuals with neither cardiopulmonary nor renal complications.

Of all acute care visits, 11,448 (62.2%) were for pain crises with 4336 hospitalizations and 7112 treat and release ED visits. Overall, there was a median of 1 acute care visit (IQR = 0–2) for pain crises. Similar to all-cause acute care utilization, there was a significantly higher proportion with 3 or more acute care visits for pain crises among the cardiopulmonary group (33%) as compared to those who had neither of the CPR end organ complications (7.8%) (p-value = 0.0002). However, acute care use was not significantly different between those with renal complications (16%) and those with no CPR end organ complications (7.8%), (p = 0.08) (Fig 3).

After adjusting for age and multiple comparisons, individuals with SCD who had CPR compilations had a significantly higher rate of acute care visits for pain crises than individuals who did not have CPR end organ complications (3.5 (IQR = 3.2–3.7) vs 1.2 (IQR– 1.1–1.2) per person per year). Similar to all cause acute care use, those with only renal complications did not have a significantly different rate of acute care use for pain crises as compared to those with no CPR complications (1.5 (IQR = 1.3–1.6) per person per year vs 1.2 (IQR = 1.0–1.3) per person per year).

## Discussion

Our study shows that almost 50% of the SCD population in Wisconsin has associated cardiopulmonary and renal complications with a variable prevalence across age groups. Overall, individuals with cardiopulmonary complications had a significantly higher rate of acute care visits (for any cause and for pain crises) as compared to those who did not have cardiopulmonary complications. There were no significant differences in acute care utilization (for any cause and for pain crises) among individuals with SCD who had renal complications compared to those who did not have renal complications, after adjusting for age.

The prevalence of cardiopulmonary complications was significantly different between age groups. The highest prevalence was seen among adults, then the transition group, with the lowest among the pediatric SCD population. The high rates of CPR end organ complications among the transition group suggest that these vital organ systems are impacted at a young age among individuals with SCD. The prevalence of the select cardiopulmonary and renal complications is much lower among the general population as compared to those with SCD. For example, prevalence of pulmonary hypertension among African-Americans has been reported as 6.8% [18], whereas in our study around 14% of individuals with SCD had pulmonary hypertension. Similarly, prevalence of chronic kidney disease in the general US population is 6% among adults 18–44 years of age [19], whereas in our study 12% of the adults with SCD had chronic kidney disease. This highlights that SCD is a multiorgan disease and individuals with SCD require multispecialty care. Future studies are needed to examine the reasons for such high prevalence of CPR complications among individuals with SCD as compared to the general population.

The differences in prevalence between age groups, especially between the pediatric and transition age groups, could be due to various reasons. It is possible that different screening practices exist between the pediatric and adult population. It is also possible that there are gaps in care for individuals during the vulnerable transition period, leading to end organ complications that emerge, and are then not appropriately managed due to these care gaps in which ultimately allows for clinical progression [20]. Future work is required to understand the risk factors for these complications for different age groups. If risk factors are identified, there would be a need to determine whether distinct screening recommendations for the pediatric population who are at risk of developing CPR end organ complications should be established. Further, guidelines for screening for CPR complications again prior to transition of from pediatric to adult health care would better inform the transition process and ensure that individuals are able to get timely care to manage their end organ complications if discovered.

Our data show acute care utilization for all-cause and/or for pain crises was significantly higher between individuals with no CPR complications and with cardiopulmonary complications after adjusting for age. This contrasted with our data that show all cause and pain crises acute care utilization for those with only renal complications were not significantly higher compared to those who did not have these complications after adjusting for age. Due to the nature of the end organ complication on of renal disease, it may be possible that renal disease alone does not result in acute clinical manifestations/exacerbations that require acute care assessment and treatment in comparison to the clinical nature of cardio-pulmonary complications that often have disease manifestations that require urgent assessment and treatment. Therefore, as reflected in our data, renal complications alone do not result in a significantly higher rate of all cause acute care utilization and acute care utilization for pain. These data suggest there may be a distinct disease phenotype in terms of acute care use among individuals with SCD who have cardiopulmonary complications, irrespective of age. It is possible that the underlying pathophysiology of cardiopulmonary complications is different from that of renal complications [21]. Although limited by small sample size, these data suggest that using acute care utilization for pain crises or any other cause does not necessarily capture the spectrum of morbidity that renal complications portend for individuals with SCD. Other important outcomes such as the patient- reported outcomes are necessary to fully understand the morbidity of this end-organ chronic complication. Further, end organ damage increases the healthcare costs of SCD by 2–5 fold, thus imposing a high economic burden both at the healthcare system and individual level [22]. Future work will focus on determining the etiology of acute care visits for reasons other than pain in individuals with SCD who have CPR complications. This will help understand the acute care needs of this subgroup of individuals having CPR end-organ damage, and potentially help design strategies to prevent it.

Our study has limitations. The cohort of individuals with SCD and existing CPR complications was identified using ICD codes. Although the ICD codes for SCD case definition are well established and validated for children and adults. The algorithm used to define the cohort of individuals with SCD is based on the number of visits and claims, which may miss individuals who do not interact as frequently with the healthcare system, may have less severe SCD and/or do not have severe complications. This algorithm may select for those that have more severe disease with increased acute care utilization which may be associated with end organ complications and overestimate the true prevalence of CPR complications. However, despite these limitations, utilizing this validated algorithm facilitates the most precise identification of those with sickle cell disease within administrative data. Another limitation of our study is that the ICD codes used to define CPR complications lack the ability to determine if these complications have been confirmed and also have not been validated for SCD. However, the complications of interest are chronic in nature and likely are specific to the complication. These codes have also been used by previously published work using administrative claims data [23, 24]. The use of these codes, however, may underestimate the prevalence of cardiopulmonary and renal complications as it will not capture those who may have these underlying conditions but are not diagnosed. Thus, collectively, these limitations could under or overestimate the true prevalence of CPR complications. Also, the cause of pain crises might not be captured accurately by ICD codes. The place of service, however, would capture all causes of utilization and does not depend on ICD codes. Data are specific to the state of Wisconsin, and the majority of individuals with SCD live in Milwaukee which has high poverty rates which could impact the generalizability of our findings. Ultimately, this could be reflected in limited access to recommended care thereby affecting the diagnosis of CPR complications and acute care utilization among the SCD population. Additional studies are needed to determine if the prevalence of the CPR complications vary across the SCD population in other states and countries. Finally, although we used linked electronic health record data from two large tertiary care facilities and administrative claims data from Medicaid and other voluntary all-payer claims database to determine acute care utilization for our population, our program does not include individuals using Medicare fee for service who do not receive care at the two tertiary care facilities. However, 85% of the state’s Black population is in the region where the tertiary care facilities are located. Finally multistate or nationwide studies with a larger cohort are needed to establish the generalizability of our findings.

## Conclusion

Almost 50% of individuals living with SCD in Wisconsin have either cardiopulmonary or renal end-organ damage. Individuals with SCD and cardiopulmonary complications have a significantly higher rate of acute care utilization, whereas renal only complications were not associated with a higher acute care utilization rate. Future work is needed to understand the distinct pathophysiology of cardiopulmonary and renal complications among individuals with SCD and the risk factors for these complications. Prospective studies are needed to determine differences in prevalence and acute care utilization between individuals that screen positive and those confirmed to have cardiopulmonary complications.

## Supporting information

S1 Table ICD codes for cardiopulmonary and renal complications included in the study. (DOCX)
