# Supplementary material for: Acute care utilization among individuals with sickle cell disease and related cardiopulmonary and renal complications
Source: PLoS One. 2024 Apr 16;19(4):e0297469. doi: 10.1371/journal.pone.0297469 (PMC11020686; doi:10.1371/journal.pone.0297469)
Supplement: S1 Table — (DOCX) [file pone.0297469.s001.docx]

| End organ complication | ICD 10 | ICD 10 description |
| --- | --- | --- |
| Heart failure |  |  |
|  | I50.9 | Heart failure, unspecified |
|  | I50.1 | Left ventricular failure, unspecified |
|  | I50.20 | Systolic (congestive) heart failure, unspecified |
|  | I50.21 | Acute systolic (congestive) heart failure |
|  | I50.22 | Chronic systolic (congestive) heart failure |
|  | I50.23 | Acute on chronic systolic (congestive) heart failure |
|  | I50.30 | Diastolic (congestive) heart failure, unspecified |
|  | I50.31 | Acute diastolic (congestive) heart failure |
|  | I50.32 | Chronic diastolic (congestive) heart failure |
|  | I50.33 | Acute on chronic diastolic (congestive) heart failure |
|  | I50.40 | Combined systolic (congestive) and diastolic (congestive) heart failure, unspecified |
|  | I50.41 | Acute combined systolic (congestive) and diastolic (congestive) heart failure |
|  | I50.42 | Chronic combined systolic (congestive) and diastolic (congestive) heart failure |
|  | I50.43 | Acute on chronic combined systolic (congestive) and diastolic (congestive) heart failure |
|  |  |  |
|  | [I50.810](https://www.icd10data.com/ICD10CM/Codes/I00-I99/I30-I5A/I50-/I50.810) | Right heart failure, unspecified |
|  | [I50.811](https://www.icd10data.com/ICD10CM/Codes/I00-I99/I30-I5A/I50-/I50.811) | Acute right heart failure |
|  | [I50.812](https://www.icd10data.com/ICD10CM/Codes/I00-I99/I30-I5A/I50-/I50.812) | Chronic right heart failure |
|  | [I50.813](https://www.icd10data.com/ICD10CM/Codes/I00-I99/I30-I5A/I50-/I50.813) | Acute on chronic right heart failure |
|  | [I50.814](https://www.icd10data.com/ICD10CM/Codes/I00-I99/I30-I5A/I50-/I50.814) | Right heart failure due to left heart failure |
|  | [I50.82](https://www.icd10data.com/ICD10CM/Codes/I00-I99/I30-I5A/I50-/I50.82) | Biventricular heart failure |
|  | [I50.83](https://www.icd10data.com/ICD10CM/Codes/I00-I99/I30-I5A/I50-/I50.83) | High output heart failure |
|  | [I50.84](https://www.icd10data.com/ICD10CM/Codes/I00-I99/I30-I5A/I50-/I50.84) | End stage heart failure |
|  | [I50.89](https://www.icd10data.com/ICD10CM/Codes/I00-I99/I30-I5A/I50-/I50.89) | Other heart failure |
| Cardiomyopathy |  |  |
|  | I42.1 | Obstructive hypertrophic cardiomyopathy |
|  | I42.2 | Other hypertrophic cardiomyopathy |
|  | I42.8 | Other cardiomyopathies |
|  | I42.4 | Endocardial fibroelastosis |
|  | I42.5 | Other restrictive cardiomyopathy |
|  | I42.8 | Other cardiomyopathies |
|  | I43 | Cardiomyopathy in diseases classified elsewhere |
|  | I42.0 | Dilated cardiomyopathy |
| Dysrhythmia |  |  |
|  | I47.1 | Supraventricular tachycardia |
|  | I47.20 | Ventricular tachycardia, unspecified |
|  | I47.9 | Paroxysmal tachycardia, unspecified |
|  | I48.91 | Unspecified atrial fibrillation |
|  | I48.92 | Unspecified atrial flutter |
|  | I49.01 | Ventricular fibrillation |
|  | I49.02 | Ventricular flutter |
|  | I46.9 | Cardiac arrest, cause unspecified |
|  | I49.40 | Unspecified premature depolarization |
|  | I49.1 | Atrial premature depolarization |
|  | I49.3 | Ventricular premature depolarization |
|  | I49.49 | Other premature depolarization |
|  | I49.5 | Sick sinus syndrome |
|  | R00.1 | Bradycardia, unspecified |
|  | I49.8 | Other specified cardiac arrhythmias |
|  | R00.1 | Bradycardia, unspecified |
|  | I49.9 | Cardiac arrhythmia, unspecified |
|  | I47.21 | Torsades de pointes |
|  | I47.29 | Other ventricular tachycardia |
|  | I47.0 | Re-entry ventricular arrhythmia |
|  | I48.0 | Paroxysmal atrial fibrillation |
|  | I48.11 | Longstanding persistent atrial fibrillation |
|  | I48.19 | Other persistent atrial fibrillation |
|  | I48.20 | Chronic atrial fibrillation unspecified |
|  | I48.21 | Permanent atrial fibrillation |
|  | I48.3 | Typical atrial flutter |
|  | I48.4 | Atypical atrial flutter |
|  | I49.2 | Junctional premature depolarization |
|  | I46.2 | Cardiac arrest due to underlying cardiac condition |
|  | I46.8 | Cardiac arrest due to other underlying condition |
| Pulmonary hypertension | [I27](https://www.icd10data.com/ICD10CM/Codes/I00-I99/I26-I28/I27-/I27) | Other pulmonary heart diseases |
|  | [I27.0](https://www.icd10data.com/ICD10CM/Codes/I00-I99/I26-I28/I27-/I27.0) | Primary pulmonary hypertension |
|  | [I27.2](https://www.icd10data.com/ICD10CM/Codes/I00-I99/I26-I28/I27-/I27.2) | Other secondary pulmonary hypertension |
|  | [I27.20](https://www.icd10data.com/ICD10CM/Codes/I00-I99/I26-I28/I27-/I27.20) | Pulmonary hypertension, unspecified |
|  | [I27.21](https://www.icd10data.com/ICD10CM/Codes/I00-I99/I26-I28/I27-/I27.21) | Secondary pulmonary arterial hypertension |
|  | [I27.22](https://www.icd10data.com/ICD10CM/Codes/I00-I99/I26-I28/I27-/I27.22) | Pulmonary hypertension due to left heart disease |
|  | [I27.23](https://www.icd10data.com/ICD10CM/Codes/I00-I99/I26-I28/I27-/I27.23) | Pulmonary hypertension due to lung diseases and hypoxia |
|  | [I27.24](https://www.icd10data.com/ICD10CM/Codes/I00-I99/I26-I28/I27-/I27.24) | Chronic thromboembolic pulmonary hypertension |
|  | [I27.29](https://www.icd10data.com/ICD10CM/Codes/I00-I99/I26-I28/I27-/I27.29) | Other secondary pulmonary hypertension |
|  | [I27.8](https://www.icd10data.com/ICD10CM/Codes/I00-I99/I26-I28/I27-/I27.8) | Other specified pulmonary heart diseases |
|  | [I27.89](https://www.icd10data.com/ICD10CM/Codes/I00-I99/I26-I28/I27-/I27.89) | Other specified pulmonary heart diseases |
|  | [I27.9](https://www.icd10data.com/ICD10CM/Codes/I00-I99/I26-I28/I27-/I27.9) | Pulmonary heart disease, unspecified |
| long qt |  |  |
|  | I45.81 | Long QT syndrome |
| Pulmonary fibrosis |  |  |
|  | J84.10 | Pulmonary fibrosis, unspecified |
| Chronic kidney disease |  |  |
|  | N18.1 | Chronic kidney disease, stage 1 |
|  | N18.2 | Chronic kidney disease, stage 2 (mild) |
|  | N18.30 | Chronic kidney disease, stage 3 unspecified |
|  | N18.31 | Chronic kidney disease, stage 3a |
|  | N18.32 | Chronic kidney disease, stage 3b |
|  | N18.4 | Chronic kidney disease, stage 4 (severe) |
|  | N18.5 | Chronic kidney disease, stage 5 |
|  | N18.6 | End stage renal disease |
|  | N18.9 | Chronic kidney disease, unspecified |
|  | N19 | Unspecified kidney failure |
| Proteinuria |  |  |
|  | R80.0 | Isolated proteinuria |
|  | R80.1 | Persistent proteinuria, unspecified |
|  | R80.8 | Other proteinuria |
|  | R80.9 | Proteinuria, unspecified |
| Hematuria |  |  |
|  | R31.0 | Gross hematuria |
|  | R31.21 | Asymptomatic microscopic hematuria |
|  | R31.29 | Other microscopic hematuria |
|  | R31.9 | Hematuria, unspecified |
